# Self-harm in British South Asian women: psychosocial correlates and strategies for prevention

**DOI:** 10.1186/1744-859X-5-7

**Published:** 2006-05-22

**Authors:** MI Husain, W Waheed, Nusrat Husain

**Affiliations:** 1 St. George's, University of London, London, UK; 2 Department of Psychiatry, The University of Manchester, Manchester, UK; 3 Lancashire Care NHS Trust, Preston, UK; 4 Department of Psychiatry, The University of Toronto, Toronto, Canada

## Abstract

**Objective:**

To review the rates of self-harm in British South Asian women, look into the factors that contribute to these high rates of self-harm and discuss possible strategies for prevention and provision of culturally sensitive service for South Asian women who harm themselves.

**Method:**

Review.

**Results:**

South Asian women are significantly more likely to self harm between ages 16–24 years than white women. Across all age groups the rates of self harm are lower in South Asian men as compared to South Asian women. These women are generally younger, likely to be married and less likely to be unemployed or use alcohol or other drugs. They report more relationship problems within the family. South Asian women are less likely to attend the ER with repeat episode since they hold the view that mainstream services do not meet their needs.

**Conclusion:**

South Asian women are at an increased risk of self harm. Their demographic characteristics, precipitating factors and clinical management are different than whites. There is an urgent need for all those concerned with the mental health services for ethnic minorities to take positive action and eradicate the barriers that prevent British South Asians from seeking help. There is a need to move away from stereotypes and overgeneralisations and start from the user's frame of reference, taking into account family dynamics, belief systems and cultural constraints.

## Introduction

Britain is a multicultural society. Nearly 6.4 million people in England belong to the ethnic minority communities. This figure represents about 1 in 8 of England's population [1]. The ethnic minority communities in England share a number of features. Disadvantage and discrimination characterise their experiences in this country in almost every aspect of life. This is particularly prevalent in the area of health and healthcare. Those from minority ethnic groups tend to suffer from poorer health, have reduced life expectancy and have greater problems with accessing health care than the majority white population. However, there have been many policy and service initiations within the National Health Service aimed at reducing ethnic variations in disease incidence, access to care and service experience [2].

Mental health is an area of particular concern for the minority communities in this country. For years, the disparities and inequalities between black and minority ethnic groups (BME) and the indigenous white population in the rates of mental illness has been the focus of concern, debate and research. Of all the mental health issues, the significantly raised risk of suicide and attempted suicide among young women of South Asian origin is of particular concern. By the year 2010, reducing the suicide rate by 20% is a key national mental health target with emphasis on South Asian women [3]. There is little evidence that such concerns have led to significant progress, whether in terms of improvement of health status or a more benign service experience and positive outcome for black and minority ethnic groups. If anything, the problems experienced by minority ethnic groups within the British mental health services may be getting worse [4].

This is very pertinent in the case of British South Asians, particularly South Asian women. National data shows that women born in the Indian subcontinent and East Africa have a 40 percent higher suicide rate than women born in England and Wales [5]. Raleigh & Balarajan [6] collected data concerning rates of suicide for the two largest ethnic minorities in England and Wales i.e. Indian and West Indian. Results show that Indian males have low Standardised Mortality Rates (73) as compared to the Indian females (115). The increased rate of suicide was largely confined to a younger age group (15–34 years), the rates being more than double of those recorded for native whites.

A history of a suicide attempt appears to be an important predictor of future suicide risk [7]. All studies apart from one have reported that the risks of self-harm and suicide attempts, as well as completed suicide, are higher in South Asian women as compared to white population [Table 1]. Young women of South Asian origin are at a high-risk for suicide, even though they may not have a previous psychiatric history [8,9]. This evidence indicates the magnitude of the problem at hand and raises the issue that deliberate self harm in South Asian women needs to be studied further. There is a desperate need for the provision of culturally sensitive and relevant services for South Asian women in distress and the appropriate preventative strategies.

**Table 1:** Rates & Precipitants of Self-harm in South Asian Women in the UK

**Author(s)**	**Method**	Rates	**Precipitants**
Burke (1976)	Retrospective case note study South Asian Males n = 24 Females n = 28	2 times the rate of South Asian men, low when compared to the general population.	Interpersonal disputes
Merrill & Owens (1986)	Crossectional patients admitted to the hospital after deliberate self harm South Asian Males n = 50 Females n = 146	3 times the rate of South Asian men, higher than UK-born females	Marital problems, arranged marriages rejections of arranged marriage proposals, cultural conflict
Neeleman et al, (1996)	Cross sectional Case notes of all patients referred to a hospital based DSH team over a six month period.	Indian females: 2.6 All Asian females (Indian, Pakistani, Bangladeshi, Chinese, & Asian others): 1.68 as compared to whites. UK born Indian females rates were 7.8 times those of UK born white females	
Bhugra et al (1999)	Crossectional (A&E, general medical, psychiatric services) South Asian Males n = 24 Females n = 65	1.6 times the rate of white women and 2.5 times the rate of South Asian men. Young Asian females (i.e. = 30 years) 2.5 times the rate of white women and 7 times the rate of South Asian men.	Gender role expectations, pressure for arranged marriage, individualisation and culture conflict
Cooper et al, (2006)	Prospective (A&E) South Asian Males n = 76 Females n = 223	Young South Asian women (16–24 years) 1.5 fold increase in risk compared to White women in the same age group. South Asian women over 5 times more likely to self-harm than South Asian men.	Relationship problems with family

In this review we will look at the rates of self-harm in British South Asian women, the factors that contribute to the high rates of self-harm in these women and possible strategies for prevention and culturally sensitive service provision for South Asian women who harm themselves.

### What is self-harm?

In recent years there has been a growing interest in the issue of self-harm and wider recognition of its existence. The increasing coverage of self-harm in the mainstream media and within clinical journals reflects this growing interest and concern. However, defining self-harm is problematic since there is no universal clinical consensus. Many different terms are used to describe self-harm. These include: 'self damaging behaviour', 'attempted suicide', 'self poisoning', 'parasuicide', 'suicide attempt', 'self mutilation', 'self injury', 'self wounding' and 'deliberate self-harm' [10].

For the purposes of this paper, the following description for deliberate self-harm will be used: "Any deliberate act with a non-fatal outcome that attempts or causes self-harm or that consists of ingesting a substance in excess of its generally recognised or prescribed therapeutic dose" [11].

### Rates of deliberate self-harm in South Asian women in the UK

Deliberate self-harm accounts for more than 170,000 hospital attendances in the UK every year [12] and it is estimated that one in ten people who deliberately harm will kill themselves [13] one in one hundred will do so within a year [14]. One of the first studies to investigate self-harm in South Asians was a retrospective study of Asian Immigrants in Birmingham by Burke [15]. The study reported that the rates of self-harm among females were twice that of males. However the overall rates were low when compared to the rates among the general population.

A decade later, a study by Merrill & Owens [8] showed that rates of attempted suicide were beginning to change in Birmingham. In the South Asian cases studied over a two-year period, it was found that females were three times more likely to present. It was also found that the overall rates for South Asian-born females were significantly (statistically) higher than that for UK-born females.

Neeleman et al, [16] surveyed case notes of all patients referred to a hospital based DSH team in London over a six month period in the year 1990. Standardized referral ratios for Indian females were 2.6 and for all Asian females (Pakistani, Bangladeshi, Chinese, Asian others) it was 1.68 as compared to white Caucasian population. In 2001 Neeleman et al, [17] further report that rates of deliberate self harm in ethnic minority groups relative to whites is low in areas of high ethnic density (suggesting protection) and high (suggesting risk) in areas of low ethnic density.

In a relatively recent study carried out in London, Bhugra et al [18] report that of all the deliberate self-harm cases studied, Asian women had the highest overall rates; 1.6 times those of white women and 2.5 times the rate among Asian men. In young Asian females (i.e. under 30 years of age) the rates were 2.5 times those of white women and 7 times those of Asian men.

Most recently a study in Manchester [9] also confirms a high population burden for self-harm in young South Asian women with rates not very different than stated elsewhere [8,18]. The rate of self-harm in young South Asian women (16–24 years) indicates a 1.5 fold increase in risk compared to White women in the same age group. South Asian women were over 5 times more likely to self-harm than young South Asian men. In contrast, the risk of self-harm in South Asian men was one third of that in White men.

All the studies mentioned indicate that rates of attempted and successful suicide are significantly higher among South Asian females particularly among the younger age group. This leads to the imminent question: Why do Asian women feel the need to harm themselves?

### Precipitants of deliberate self-harm in South Asian women

Historically, the reasons for killing or harming oneself vary with cultures and societies. Suicide and deliberate self-harm were common in ancient European cultures where women used hanging and men used various tools to harm themselves. According to ancient Hindu texts, suicide was permitted on religious grounds as death was seen as the beginning of another life [19]. In Islam suicide is prohibited and there has been till recently lower rates reported in the Muslim countries where it is considered to be a criminal offence [20]. Parasuicide (i.e. attempted suicide or deliberate self-harm) may be an attempt to seek help or an unsuccessful attempt to die. The act may be influenced by single or multiple stresses [19]. The relatively high rates of self-harm in South Asian women could arise due to a number of precipitating factors. These factors can range from social, political and economic pressures to domestic violence, poverty, language problems, health and family and children's issues [21].

One major precipitating factor in South Asian Women who harm themselves is marital problems. In a study by Merrill and Owens [8], in Birmingham, UK, South Asian women reported marital problems more frequently and the majority of these problems were due to cultural conflicts. A few of the Asian women in the study reported that their husbands demanded them to behave in a less westernised fashion. Also, they reported that their mother in laws interfered with the way they ran their lives and marriages. Such factors, along with arranged marriages, rejections of arranged marriage proposals and other marital problems place pressure on South Asian women, and thus were reported as precipitating factors for self-harm by the participants.

In 1999, Bhugra et al [19] compared two groups of South Asian women to study various cultural and social factors associated with attempted suicide in South Asians. From the study, it was found that those attempting suicide were more likely to have history of a past psychiatric disorder, more likely to repeat the attempt and more likely to be in an interracial relationship. It was also found that those South Asian women attempting suicide were more likely to have changed religion and spent less total time with their families. In the same study, when South Asians attempting suicide were compared with white attempters it was found that South Asians were more likely to have no psychiatric disorder, were less likely to have used alcohol in their attempt, and were more likely to have been assaulted verbally or physically. However, the findings of this study should be interpreted with caution since only a small number of individuals were interviewed.

In the study in Manchester [9] higher proportion of South Asians (particularly women) cited an interpersonal problem with family members, as the main precipitant of the self-harm episode and a higher proportion were married despite being younger.

Some of the social and cultural factors that influence rates of self-harm in South Asian women are summarised in Table 1.

The high rates of self-harm displayed by South Asian women is not a trend that is displayed by South Asian adolescents; in a study of South Asian female adolescents Bhugra et al [21] reported that rates of attempted suicide among teenagers were no different from their white counterparts. Nonetheless, South Asian female adolescents were more likely to report a family history of suicide and were more likely to recognise a cultural conflict. Otherwise white and South Asian female adolescents (aged 16–17 years) had similar adjustment reactions, alcohol and drug use, peer and relationship problems.

Kingsbury [22], in a study of adolescents who had taken overdoses showed that social and parental relationships were a key cause of isolation and as a result, attempted suicide. He found that South Asian adolescents had fewer problems with boy or girlfriends and were more likely to have problems with siblings. It was also reported that South Asian adolescents were less likely to be in contact with their friends, saw them less frequently and for shorter periods, and their relationships with their parents did not compensate for this. A school based self report survey [23] of deliberate self harm carried out in England also show that 6.7% of Asian girls as compared to 11.6% of white girls had reported self harming. Among the boys 2.7% Asian and 3.3% whites reported such behaviour.

Most South Asian communities maintain their traditional cultural identity and place great importance on academic and economic success, the stigma attached to failure, the overriding authority of elders and an unquestioning compliance from the younger members. Such cultural attitudes place hard-to-meet expectations on Asian youth leading to increased pressure and stress.

As South Asian female adolescents grow older, the rates of self-harm increase; particularly the rates of self-harm for Asian females aged 18–24 are significantly higher [9,21]. This suggests that they come under more stress. The stress may relate to gender role expectations, pressure for arranged marriage, individualisation and cultural conflict, which may precipitate attempts of self-harm.

A qualitative study of South Asian women in Manchester [24] found that issues such as racism, stereotyping of Asian women, Asian communities, and the concept of *"izzat" *(honour) in Asian family life all led to increased mental distress. The women in this study saw self-harm as a way to cope with their mental distress.

The concept of *izzat *(i.e. honour/respect) is a major influence in Asian family life. According to the women in the study, *izzat *was pervasive and internalised and it prevented other community members from listening and getting involved. The burden of *izzat *was unequally placed upon the women in Asian families and as a result this created hard-to-achieve high expectations of women as daughters, daughters-in-law, sisters, wives and mothers.

Furthermore, many Asian families are critical about the behaviour of women and it is very important whether this is seen as 'good' behaviour according to the community since it is essential in gaining status and prestige for the family. The women in the study reported that a community grapevine often develops in Asian communities in the UK due to this. This grapevine then results in a lack of privacy and space for women. Many women in the study felt as though they had nobody to trust and thus could not speak to anyone in the community. This leads to an increasing sense of isolation for Asian women.

All of the participants in the study mentioned above stated that they would not be able to access mainstream service provision because they would not be able to trust the providers of these services. The fear of the community grapevine even prevents these women from seeking help from their GP's. They feared that 'the GP might be your family GP and might tell your parents' or 'it would go down on your record'. Also the women feared that the General Practice staff or the GP might be part of the local community. As stated before, these women already have a feeling of isolation, the barriers to accessing GP's and other service providers add to this feeling of isolation.

The participants in the study also agreed that an inability to speak English increased their sense of isolation. Those who had resorted to self-harm reported that problems at school; bullying, including racist bullying; forced marriage; domestic violence; migration and loss of culture and family; problems with in-laws; children; health; and not having a confiding relationship as the precipitating factors for their behaviour. The women used self-harm as a response to social isolation and as logical behaviour to reduce distress and ask for help.

In a majority of South Asian women presenting with attempted suicide, self-harm was seen as a last resort, but as a logical response to extreme distress [24].

### Strategies for management, intervention and prevention

As mentioned before, reduction in the number of suicides is a central theme in the government's Health of the Nation strategy for England. However, there is a considerable lack of knowledge as to which preventive strategies are effective [25].

Hawton et al [25] used the repetition of deliberate self-harm as an alternative measure to investigate the effectiveness of different intervention strategies. Promising results were found for problem solving therapy, provision of a card to allow patients to make emergency contact with services, flupenthixol for recurrent self harm. It was also found that assertive outreach can help to keep patients in treatment. Furthermore, Guthrie et al [26] found that compared with usual treatment, four sessions of psychodynamic interpersonal therapy reduced suicidal ideation and self-reported self harm. Cognitive behavioural therapy is also a promising method that could possibly be used in the management of deliberate self-harm [27-29].

In the South Asian context a recurrent theme within the qualitative studies is that the survivors of suicide attempts do not feel "heard" or understood either by their families or by mental health workers [30]. Chew-Graham [24] found that South Asian women were apprehensive to access mainstream service provision because they would not be able to trust the providers of these services and would only access support in cases of extreme crises. Thus, work needs to be carried out to help agencies build trust with South Asian women.

In the study in Manchester [9] South Asians were more likely to be assessed by accident and emergency staff (and less likely to be assessed by a mental health specialist) than Whites, although these differences were statistically significant only in women. Overall, clinical staff tended to rate both South Asian men and women as being at lower medical risk and lower risk of future self-harm compared to Whites. South Asians of both sexes were more likely to be discharged from emergency department without referral to other services, and be referred to their GP (either by letter or told to see), and they were less likely than Whites to be referred to specialist medical, surgical or psychiatric services.

Cultural barriers also prevent South Asian women from accessing support. In the study by Chew-Graham [24] women stated that the service providers were usually white and lacked understanding of Asian culture. The women felt as though they would be judged by people who had fixed views about the Asian community and that they would offer simplistic yet unrealistic solutions like 'leaving the family' without understanding the complexity of the situation. The women in the study gave their suggestions on how to improve service provision for South Asian women in distress. These suggestions included advertising services and raising awareness about what is meant by 'psychology', 'counselling' or 'mental health' in places where the Asian community were located. The production of translated information leaflets was another suggestion. Some of the women stated that they would want counsellors of the same background as them while some women were highly opposed to this because of fear of the community grapevine. Other suggestions for improving services also included: advertising them in places where Asian women could access them, especially if they could not read/speak English; providing services in schools to young Asian women; running local groups; training of health visitors to provide information to young mothers on services; Urdu leaflets and raising awareness on mental health, service provision and access.

There have been many studies addressing the sociodemographic variables, help seeking and need for culturally appropriate services for South Asian community in the UK however, very little is known about interventions. We have only found one published study by Bhugra & Hicks [31] which has reported positive impact on help seeking attitudes for depression and suicidality in South Asian women with the intervention using a simple educational pamphlet. Further Studies are needed in this area in order to come up with an effective method of prevention.

The key to developing effective prevention strategies is to employ them at the right time and make them culturally sensitive [32]. With no differences in suicide rates in adolescents and then a sudden rise in the rates among young women, this offers us a chance to focus on this window of opportunity. Further research into risk and protective factors at this level can guide us in developing our preventive interventions.

Secondary prevention can also be achieved by addressing interventions in depressed South Asian women who have comparatively higher prevalence of depression than Whites [33]; this can indirectly help in lowering rates of self harm. Active psychosocial management of persistent stress factorsin self harm repeaters is another high priority area which can help in reducing overall mortality.

## Conclusion

This review studied self-harm in South Asian women. We find that South Asian women are at a significantly higher risk of self-harm than white European women. This paper also provides a list of possible precipitating factors and analyses the factors that drive Asian women in the UK to harm themselves. Nonetheless this behaviour is seen as the last resort.

Since Burke [15] first examined self-harm in South Asian immigrants in 1976, there have been a number of studies in this area. Nonetheless we have found only one published study on interventions for South Asian women who harm themselves in the UK. There are a number of different approaches being used to overcome the threat of self-harm in the white population, as yet there is no firm recommendation for the treatment and prevention of deliberate self-harm in South Asians.

One possible approach is to look at South Asian groups individually on the basis of their national identity and religious affiliation. In the majority of studies concerning self-harm in Asians, the author(s) did not consider the diversity that exists within the South Asian community. Diversity in South Asian communities is seen primarily in terms of national identity (country of their family origin) or religious affiliation. Therefore diversity within South Asian communities is mentioned in terms of Pakistani, Bangladeshi, and Indian etc. In most studies concerning self-harm and Asians, Pakistani, Indian and Bangladeshi subjects were all generally placed under the same category of South Asian. However, differences between each of these groups in terms of language, religion and economic circumstances do exist. The details of specific differences within each minority ethnic group should be examined before services can be appropriately tailored [10]. Furthermore, each of the groups (i.e. Pakistani, Bangladeshi etc.) should be investigated individually in order to obtain a more accurate picture of the problem at hand.

This paper indicates the urgent need for all those concerned with the mental health services for ethnic minorities to take positive action and eradicate the barriers that prevent South Asians from seeking help. There is a need to move away from stereotypes and overgeneralisations and start from the user's frame of reference, taking into account family dynamics, belief systems and cultural constraints.

The key to developing prevention strategies is to employ them at the right time and make them culturally sensitive to be effective. With no differences in suicide rates in adolescents and then sudden rise in young women offers us a chance to focus on this window of opportunity. Research to look at risk and protective factors at this level can guide us in developing our interventions. These can include School based health education programmes and community based health education programmes like working with print, electronic media, voluntary and religious organisations.

There is now enough evidence base for secondary prevention in the general population the immediate action required is to culturally adapt the content and delivery mechanism to address this major public health unmet need.
